# *Exophiala jeanselmei* Lower Extremity Phaeohyphomycosis in a Solid Organ Transplant Recipient

**DOI:** 10.4269/ajtmh.26-0397

**Published:** 2026-10-01

**Authors:** Ashton D. Hall, Deepali Boothankad Sharath, Anisha Misra, Steven D. Billings, Jessica Lum

**Affiliations:** ^1^ Department of Internal Medicine, Primary Care Institute, Cleveland Clinic, Cleveland, Ohio, USA;; ^2^ Department of Infectious Diseases, Integrated Hospital Care Institute, Cleveland Clinic, Cleveland, Ohio, USA;; ^3^ Department of Laboratory Medicine, Robert J. Tomsich Pathology and Laboratory Medicine Institute, Cleveland Clinic, Cleveland, Ohio, USA;; ^4^ Department of Pathology, Robert J. Tomsich Pathology and Laboratory Medicine Institute, Cleveland Clinic, Cleveland, Ohio, USA

A 65-year-old man with a nonsequential liver and kidney transplant who was on tacrolimus, prednisone, and mycophenolate mofetil developed a painless, raised nodular lesion on his left heel 2 years after transplantation. He was an avid gardener who wore open-heeled shoes. Three months later, the lesion became painful with serosanguineous drainage, prompting medical evaluation (Figure 1). The initial wound culture grew coagulase-negative staphylococci; however, trimethoprim–sulfamethoxazole failed to improve his symptoms.

**Figure 1.**
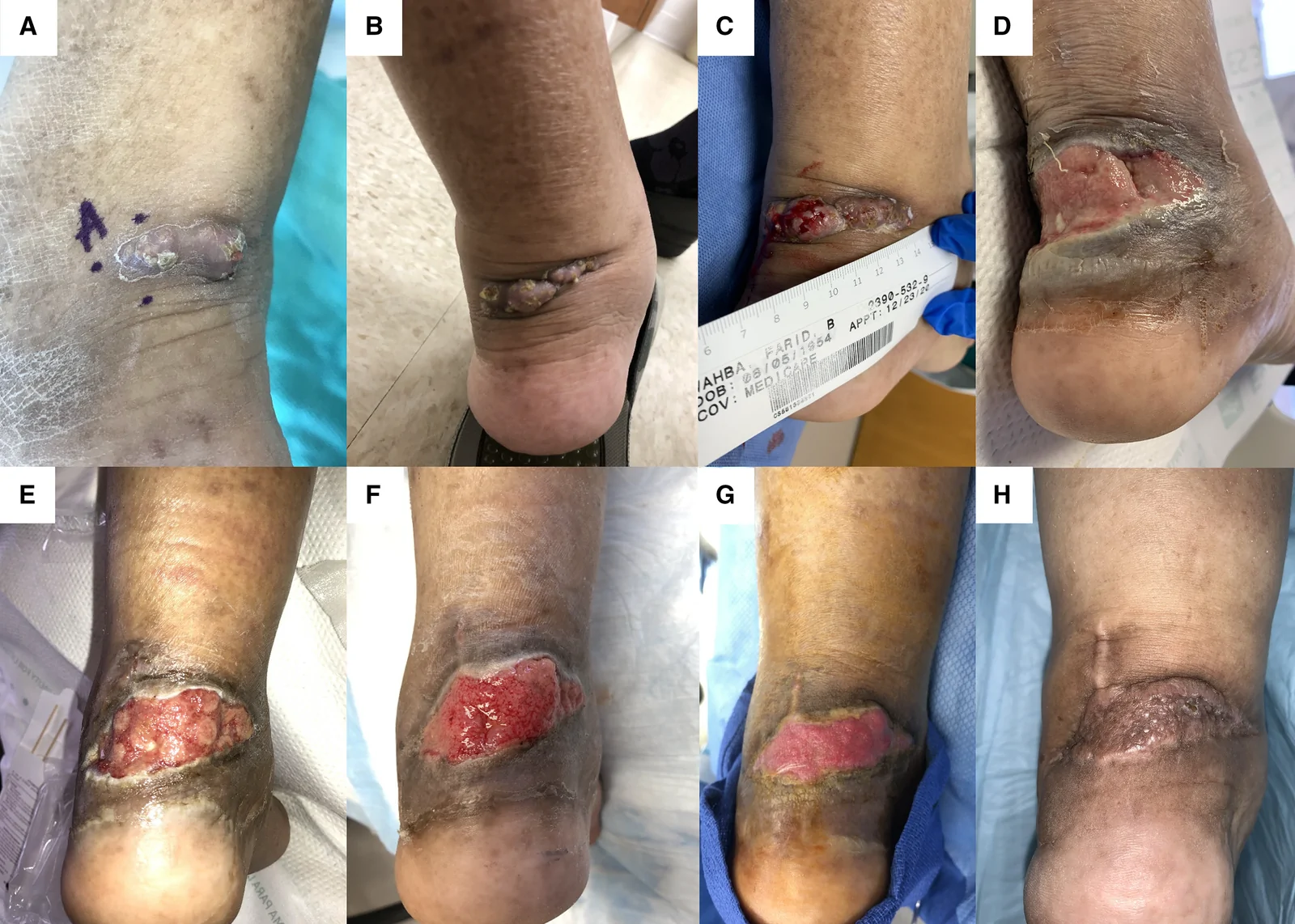
Eight photographs of the patient’s left heel. (**A**) At presentation, (**B**) 1 month later, and (**C**) 2 months later, the painful, rough, nodular lesion progressively enlarged. After surgical excision, the wound is shown at (**D**) 2 weeks, (**E**) 4 weeks, (**F**) 6 weeks, and (**G**) 12 weeks, immediately before split-thickness skin grafting. (**H**) The final image reveals a favorable outcome 2 months after skin grafting.

The patient underwent surgical excision of lesions on the left heel and calf (Figure 2). Histopathological examination revealed suppurative granulomatous inflammation with fungal hyphae. Grocott-Gömöri’s methenamine silver (GMS) staining revealed septate hyphae with acute-angle branching, and a fungal culture grew *Exophiala jeanselmei* (Figure 3). Postoperative magnetic resonance imaging revealed inflammatory soft tissue changes with nodular lesions near the Achilles tendon but without bone involvement (Figure 4). Two weeks later, the patient underwent a second debridement involving the subcutaneous tissue, Achilles tendon, and paratenon. A fungal culture grew dematiaceous mold.

**Figure 2.**
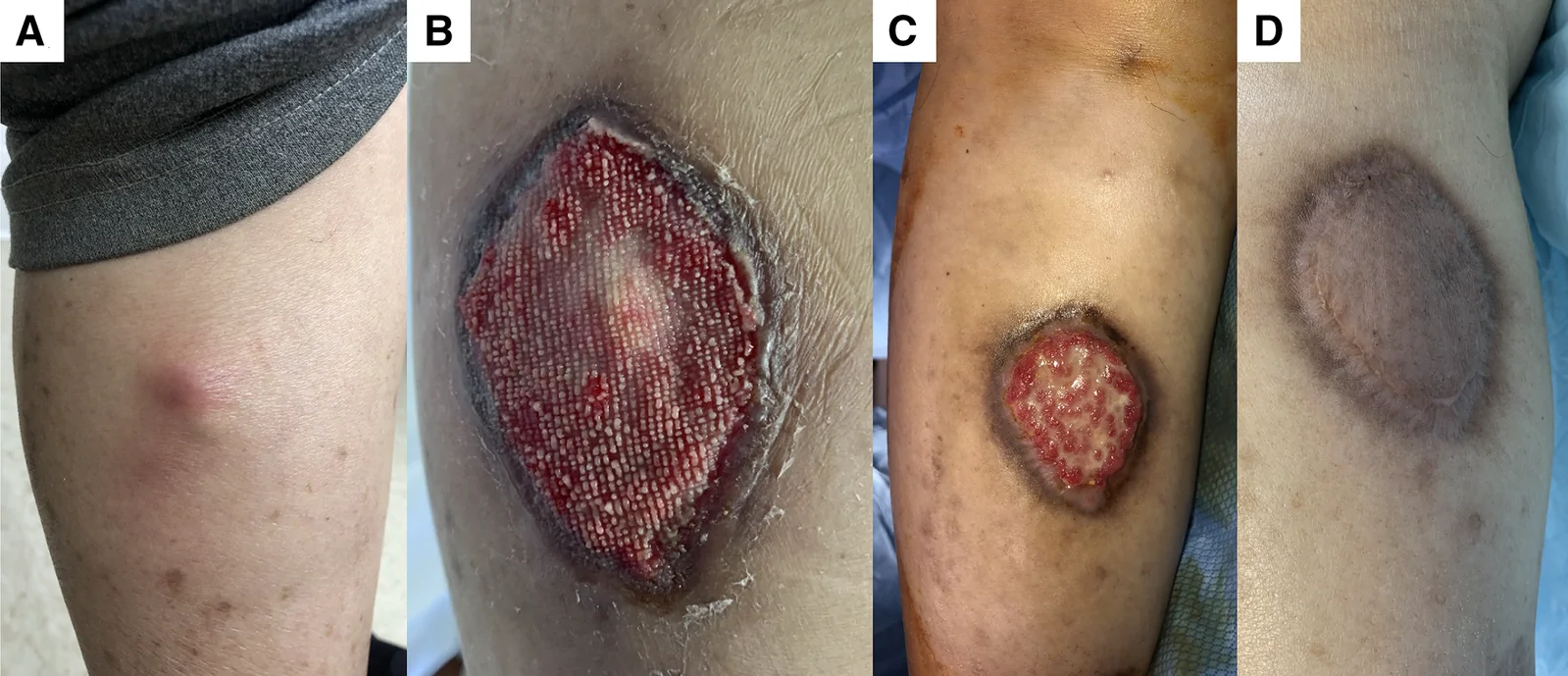
Four images of the patient’s left calf. (**A**) A nodular lesion 1 month after presentation. (**B** and **C**) The postoperative wound at 2 weeks and 12 weeks after surgical excision. (**D**) The calf 2 months after spit-thickness skin grafting with complete wound healing.

**Figure 3.**
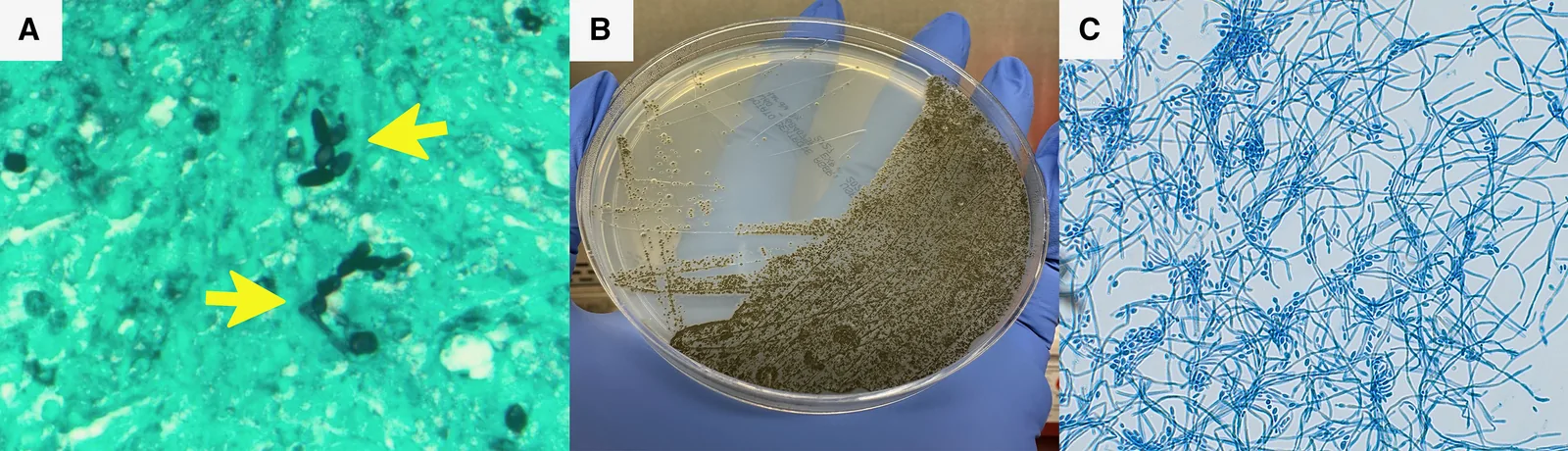
Morphological characteristics of *Exophiala* species. (**A**) Grocott-Gömöri’s methenamine silver stain of tissue from the patient’s initial left heel excision exhibits numerous fungal hyphae (yellow arrows). (**B**) Macroscopic appearance on potato dextrose agar at 5 days. The colony is slow-growing, initially smooth and mucoid-black, later developing a raised, velvety, olivaceous-black texture with a solid black reverse. (**C**) Microscopic features of *Exophiala* species via lactophenol cotton blue stain at 400× magnification. Highly septate, pigmented hyphae are shown alongside budding yeast-like cells. Cylindrical, tapering annellides produce clusters of small, single-celled, hyaline conidia at their apices.

**Figure 4.**
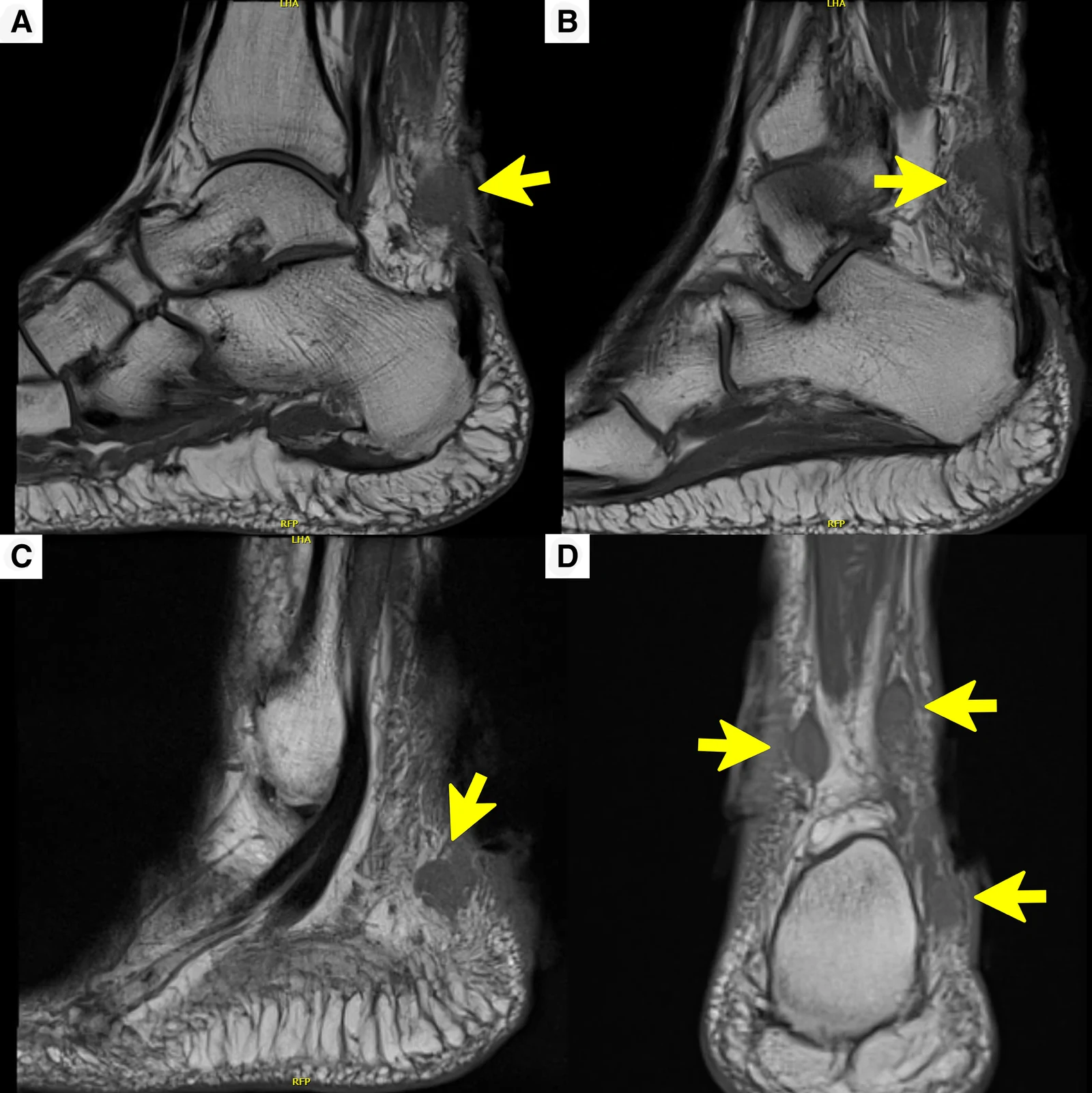
Magnetic resonance imaging of the left ankle 1 day after the patient’s initial debridement, exhibiting postoperative and inflammatory changes in the soft tissues of the posterior ankle near the Achilles tendon without involving the bone. Three nodular areas are present around the ankle (yellow arrows), measuring up to 1.7 × 1.5 cm. The patient underwent a repeat debridement 2 weeks later.

Antifungal susceptibility testing was performed at the University of Texas at San Antonio. Posaconazole (minimum inhibitory concentration [MIC] ≤0.03) was the preferred agent, but it was cost-prohibitive; therefore, itraconazole (MIC ≤0.06) was initiated at a dose of 200 mg every 12 hours. Given the concern for drug–drug interaction with itraconazole, the tacrolimus dose was reduced by 50%. Because of residual infection adherent to the fascia, the patient was admitted for 7 days of amphotericin dwellings via a vacuum-assisted closure device (15 mL for 10 minutes every 1.5 hours). Persistently subtherapeutic itraconazole levels and dose-limiting side effects prompted the addition of terbinafine (500 mg/day), followed by transition to posaconazole (300 mg/day) plus terbinafine (250 mg/day). Therapeutic posaconazole levels (1.0 to 1.3 *µ*g/mL) were achieved. The patient completed at least 13 months of antifungal therapy, including 6 months of combination therapy and 7 months of posaconazole monotherapy. Split-thickness skin grafting was performed 2 months after the second debridement. Four years after completing treatment, he remains free of recurrent infection.

Dematiaceous fungi, particularly *Exophiala* species, are a common cause of phaeohyphomycosis.^1^^,^^2^ Implantation mycoses, including phaeohyphomycosis, can develop when environmental fungi are introduced into the skin and subcutaneous tissues through penetrating injuries from thorns, wood splinters, or gardening tools, particularly in immunocompromised patients.^1^^–^^3^ Phaeohyphomycosis may remain confined to the skin and subcutaneous tissues or involve the lungs, central nervous system (CNS), or disseminate.^1^^,^^4^ Diagnosis relies on fungal culture and histopathology with periodic acid-Schiff, Fontana-Masson, or GMS staining.^2^^,^^5^ The differential diagnosis includes chromoblastomycosis, sporotrichosis, cutaneous blastomycosis, and nontuberculous mycobacterial infections, as well as mycetoma, lobomycosis, and verrucous cutaneous leishmaniasis in tropical and subtropical regions.^6^ European guidelines recommend surgical excision with or without prolonged antifungal therapy, most commonly itraconazole, voriconazole, or posaconazole, with variable in vitro susceptibility, or combination treatment with terbinafine.^5^ Outcomes depend on the infection site, with skin and soft tissue infections carrying a more favorable prognosis than pulmonary, CNS, or disseminated disease.^1^^,^^4^
